# Liver X Receptors Suppress Activity of Cholesterol and Fatty Acid Synthesis Pathways To Oppose Gammaherpesvirus Replication

**DOI:** 10.1128/mBio.01115-18

**Published:** 2018-07-17

**Authors:** P. T. Lange, C. Schorl, D. Sahoo, V. L. Tarakanova

**Affiliations:** a Department of Microbiology and Immunology, Medical College of Wisconsin, Milwaukee, Wisconsin, USA; b Department of Molecular Biology, Cell Biology and Biochemistry, Brown University, Providence, Rhode Island, USA; c Division of Endocrinology, Metabolism and Clinical Nutrition, Department of Medicine, Medical College of Wisconsin, Milwaukee, Wisconsin, USA; d Cardiovascular Center, Medical College of Wisconsin, Milwaukee, Wisconsin, USA; e Cancer Center, Medical College of Wisconsin, Milwaukee, Wisconsin, USA; Stony Brook University

**Keywords:** cholesterol synthesis, fatty acid synthesis, gammaherpesvirus, liver X receptors, macrophages

## Abstract

Gammaherpesviruses are oncogenic pathogens that persist in ~95% of the adult population. Cellular metabolic pathways have emerged as important regulators of many viral infections, including infections by gammaherpesviruses that require several lipid synthetic pathways for optimal replication. Liver X receptors (LXRs) are transcription factors that are critical regulators of cellular fatty acid and cholesterol synthesis pathways. Not surprisingly, LXRs are attractive therapeutic targets in cardiovascular disease. Here we describe an antiviral role for LXRs in the context of gammaherpesvirus infection of primary macrophages. We show that type I interferon increased LXR expression following infection. Surprisingly, there was not a corresponding induction of LXR target genes. Rather, LXRs suppressed the expression of target genes, leading to decreased fatty acid and cholesterol synthesis, two metabolic pathways that support gammaherpesvirus replication. This report defines LXR-mediated restriction of cholesterol and lipid synthesis as an intrinsic metabolic mechanism to restrict viral replication in innate immune cells.

## IMPORTANCE

Fatty acid and cholesterol synthesis pathways of the host play important roles in diverse biological systems. Importantly, these two metabolic pathways are also usurped by a number of viruses to facilitate viral replication. In this report, we show that suppression of these pathways by liver X receptors in primary macrophages creates an intrinsic antiviral state that attenuates gammaherpesvirus replication by limiting viral access to the two metabolic pathways.

## INTRODUCTION

Liver X receptors (LXRs) are members of the nuclear receptor transcription factor family that are activated by ligands to mediate expression of target genes (1). LXRα and LXRβ are two related LXR isoforms with tissue-specific expression and ubiquitous expression, respectively. Consistent with the role of LXRs as metabolic regulators, most LXR target genes encode proteins that mediate fatty acid synthesis or cholesterol efflux from cells. Not surprisingly, LXRs have received much attention as therapeutic targets, especially in cardiovascular disease.

Metabolic pathways of the host orchestrate replication of diverse viruses. The ability of LXRs to regulate expression of proteins and enzymes involved in lipid metabolism is, therefore, likely to modify viral replication. Intriguingly, in the few published virus-host interaction studies, LXRs have been reported to play opposing roles in viral replication. Activation of LXRs stimulates expression of fatty acid synthesis enzymes and subsequent increases in levels of intracellular fatty acids, a process that is likely to facilitate viral replication. Indeed, increased fatty acid synthesis induced by treatment with synthetic LXR agonists facilitates replication of coxsackie B virus (2).

However, active LXRs also upregulate transporters, such as ABCA1, that mediate efflux of free or unesterified cholesterol (3–5). This efflux eventually leads to depletion of intracellular cholesterol, which subsequently activates endogenous cholesterol synthesis to restore cholesterol homeostasis. Because distinct viruses have various rates of replication and differential requirements for cholesterol itself versus other intermediates of the cholesterol synthesis pathway, it is not surprising that LXR-dependent changes in the cholesterol homeostasis can have antiviral or proviral effects depending on the particular virus infection. Synthetic LXR agonists restrict replication of Newcastle disease virus (NDV) (6), hepatitis C virus (HCV) (7, 8), and human immunodeficiency virus (HIV) (9), presumably by altering cholesterol homeostasis, although the activity of cholesterol synthesis pathway was not examined in those studies. The mechanisms by which LXRs regulate viral replication remain poorly defined. To date, the majority of studies exploring LXR-virus interactions relied on artificial agonists that activate both LXR isoforms in addition to off-target effects. Further, there is little to no understanding of how LXRs regulate replication of DNA viruses.

The current report focuses on gammaherpesviruses, ubiquitous pathogens that are associated with a wide range of malignancies (10). The Lagunoff group has elegantly demonstrated that the replication and latency of Kaposi’s sarcoma-associated herpesvirus (KSHV) are supported by the fatty acid synthesis pathway (11, 12). We have shown that the related murine gammaherpesvirus 68 (MHV68) usurps intermediates of the cholesterol synthesis pathway to facilitate viral replication (13). The results of those studies suggest that, similarly to other viruses, gammaherpesviruses benefit from active cholesterol and fatty acid synthesis pathways that are also classically stimulated by LXRs. Further, 25-hydroxycholesterol (25HC), an endogenous LXR agonist (14), accumulates in MHV68-infected primary macrophages (15) and should stimulate expression of LXR target genes, with subsequent activation of metabolic pathways that support viral replication.

Surprisingly, we found that MHV68 replication was increased in primary macrophages deficient in both LXR isoforms. Consistent with this antiviral role of LXRs, expression of both the α and β isoforms was increased in infected macrophages in a type I interferon (IFN)-dependent manner. In contrast to the well-defined role of LXRs in activating expression of cholesterol efflux and fatty acid synthesis genes, LXR target genes were expressed to a lower level in wild-type macrophages than in LXR-deficient infected macrophages, along with suppressed activity of the cholesterol and fatty acid synthesis. Thus, LXR-mediated repression of metabolic pathways that are beneficial for viral replication contributed to the intrinsic antiviral state of primary innate immune cells.

## RESULTS

### LXRs attenuate MHV68 replication in primary macrophages.

**FIG 1:**
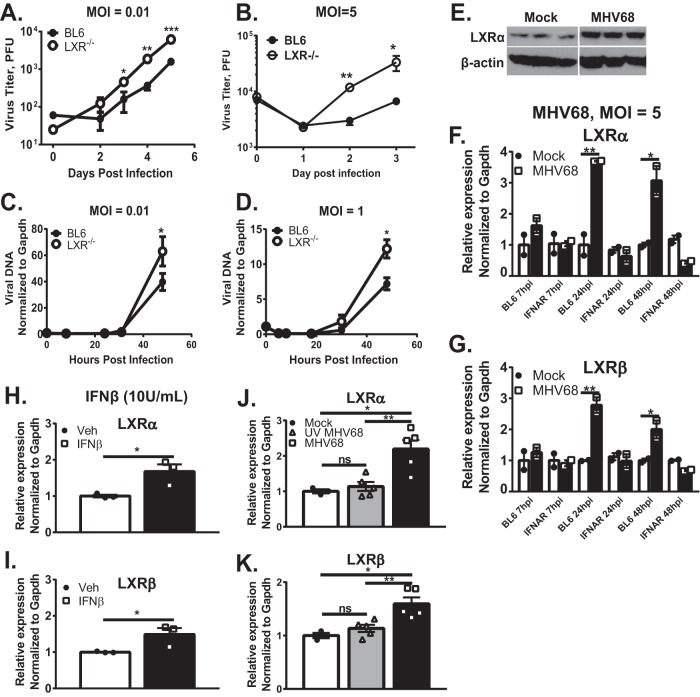
LXRs attenuate MHV68 replication in macrophages, which show increased LXR expression in a type I IFN-dependent manner. Bone marrow-derived macrophages (BMDM) of indicated genotypes were infected at an MOI of 0.01 (A and C), 5 (B, E, F, G, J, and K), or 1 (D) for the indicated time or for 24 h. Data represent total viral titers (combined cell associated and extracellular) (A and B), relative viral DNA levels (cell associated) (C and D), protein levels of LXRα and β-actin in triplicate (E), and mRNA levels of LXRα and LXRβ (F to I). The BL6 macrophages represented in panels H and I were mock treated or treated with10 U/ml of IFN-β for 24 h. (J and K) BL6 macrophages were infected with MHV68 or UV-inactivated MHV68 or were mock infected for 24 h. Expression of LXRα and LXRβ was assessed by qRT-PCR. Data shown are representative results from 2 to 4 independent experiments. Error bars represent standard errors of the means here and in the other figures. *, *P* < 0.05; **, *P* < 0.01; ***, *P* < 0.001; ns, not significant (here and in the other figures). Veh, vehicle.

To define the role of LXRs during MHV68 infection, viral replication was assessed in primary macrophages derived from C57BL/6J (BL6) mice or mice genetically deficient in both LXRα and LXRβ isoforms (16) (referred to here as LXR^−/−^ mice). Relative to BL6 macrophages, MHV68 replication was increased in LXR^−/−^ macrophages, independently of the multiplicity of infection (MOI) (Fig. 1A and B). In order to identify the stage of the viral replication cycle attenuated by LXRs, viral DNA accumulation was quantified over the course of a single MHV68 replication cycle (~48 h). Viral DNA levels were increased in LXR^−/−^ macrophages at the late stages of viral replication cycle (Fig. 1C and D). In summary, genetic deficiency of both LXR isoforms facilitated MHV68 replication, in part via enhancing late stages of the replication cycle.

### Type I interferon (IFN) is necessary and sufficient to increase LXR expression in primary macrophages.

Having observed increased MHV68 replication in LXR^−/−^ macrophages, LXR expression was assessed next. LXRα protein levels were elevated in macrophages infected with MHV68 for 24 h (Fig. 1E). A corresponding increase in the expression of both LXR isoforms was also observed at the mRNA level (Fig. 1F and G), suggesting that increased transcription was responsible for higher protein expression. Interestingly, increased mRNA levels of LXRα and LXRβ were not observed in MHV68-infected primary macrophages deficient in the IFN-α/β receptor (IFNAR^−/−^) (Fig. 1F and G), suggesting that type I IFN signaling is necessary to increase expression of LXRs during MHV68 infection.

In order to determine if type I IFN is sufficient to stimulate LXR expression, naive macrophages were treated with exogenous IFN-β concentrations similar to those induced by MHV68 infection (17). IFN-β treatment increased the expression of both LXRα and LXRβ in uninfected cells (Fig. 1H and I), suggesting that type I IFN signaling was sufficient to induce expression of either LXR isoform in primary macrophages.

We have previously shown that infection of primary macrophages with UV-inactivated MHV68 induces lower peak levels of type I IFN signaling that return to baseline by 24 h postinfection (17). In contrast, infection of primary macrophages with the equivalent amount of live MHV68 induced a higher level of type I IFN signaling that was sustained throughout the 48 h of replication (17). To determine the extent to which the host response to inactivated MHV68 was sufficient to increase LXR expression, LXR mRNA levels were compared in primary macrophages infected with either live or UV-inactivated MHV68. Expression of either LXR isoform remained at the baseline level following infection with UV-inactivated MHV68 (Fig. 1J and K), indicating that sustained type I IFN signaling in response to live MHV68 was required for increased expression of LXR. In summary, type I IFN was necessary and sufficient to induce expression of both LXR isoforms; sustained type I IFN signaling was necessary for such an increase in MHV68-infected primary macrophages.

### 25-Hydroxycholesterol (25HC) is not required for LXR expression and suppresses MHV68 replication in an LXR-independent manner.

**FIG 2:**
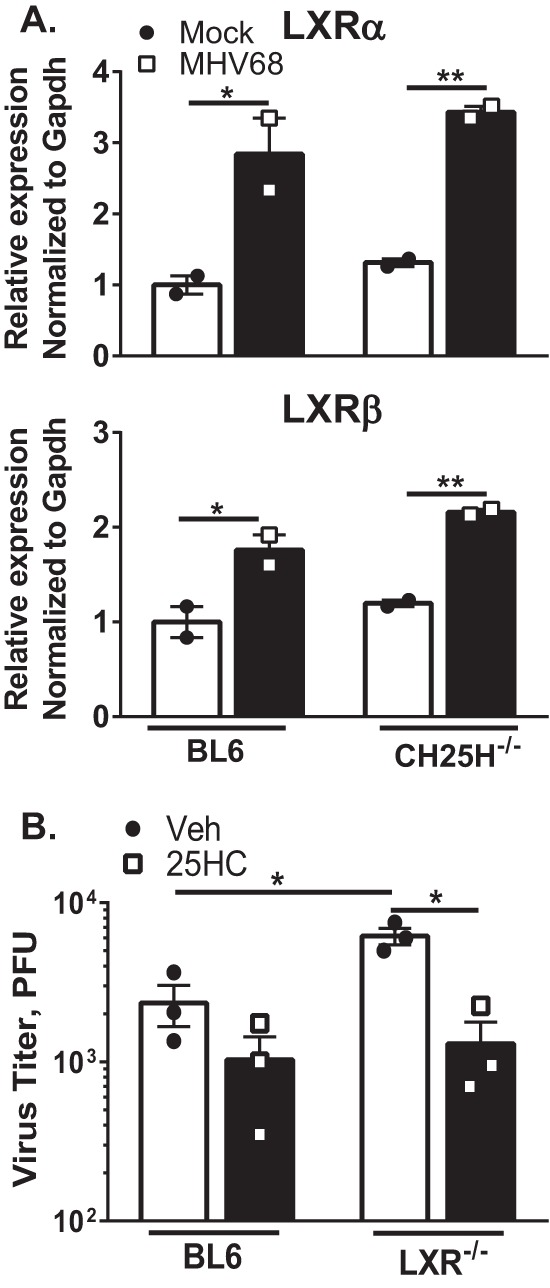
25-Hydroxycholesterol (25HC) is not required for LXR expression and suppresses MHV68 replication in an LXR-independent manner. (A) Primary macrophages derived from BL6 and CH25H^−/−^ mice were infected at an MOI of 5 for 24 h. LXRα and LXRβ expression was measured by qRT-PCR. (B) Primary macrophages derived from BL6 and LXR^−/−^ mice were infected at an MOI of 0.01 and then immediately treated with 2 µM 25HC or vehicle control for 72 h. Data shown are representative results from at least two independent experiments.

Cholesterol-25-hydroxylase (CH25H) is a unique mammalian enzyme that converts cholesterol to 25HC, an oxysterol with broad antiviral activity (18, 19). CH25H expression is increased in MHV68-infected macrophages in an interferon regulatory factor 1 (IRF-1)-dependent manner, with subsequent accumulation of 25HC (15). Because 25HC is a known LXR agonist (14) and because LXRs can increase their own expression in human macrophages (20), we asked whether 25HC was responsible for the increased LXRα and LXRβ mRNA levels seen during MHV68 infection. However, similar increases in LXRα and LXRβ mRNA levels were observed in MHV68-infected BL6 and CH25H^−/−^ macrophages (Fig. 2A). Thus, the type I IFN-dependent increase in expression of LXR isoforms was independent of CH25H.

The viral phenotypes observed in LXR^−/−^ macrophages (Fig. 1A to D) were similar to the published MHV68 phenotypes detected in CH25H^−/−^ macrophages (15), suggesting that 25HC might enact its antiviral activity by acting as an LXR agonist. To determine if LXR expression is required for the antiviral activity of 25HC, the levels of antiviral activity of 25HC were compared in BL6 and LXR^−/−^ macrophages. 25HC treatment decreased MHV68 replication to similar levels regardless of the presence or absence of LXR expression (Fig. 2B). Thus, the ability of 25HC to suppress MHV68 replication in primary macrophages was not dependent on LXR expression. In summary, expression of CH25H and its product was not required for increased LXR expression. Further, antiviral effects of 25HC did not depend on the presence of LXRs, indicating that CH25H and LXR play independent roles in suppression of MHV68 replication.

### Increased expression of LXRs in MHV68-infected macrophages does not produce a corresponding increase in the expression of LXR target genes.

**FIG 3:**
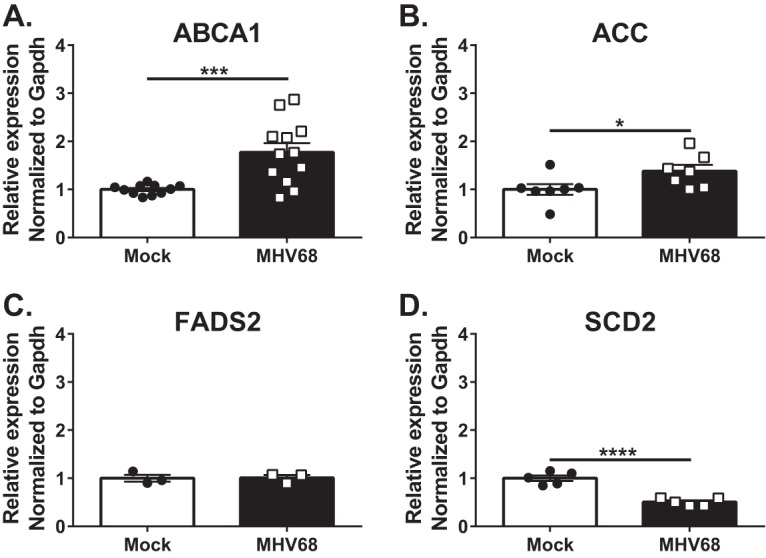
Increased expression of LXRs in MHV68-infected macrophages does not produce a corresponding increase in the expression of LXR target genes. BL6 BMDM were mock treated or infected with MHV68 at an MOI of 5 for 24 h. mRNA levels of the indicated genes were measured by qRT-PCR. Data were pooled from at least two independent experiments.

Because expression of both LXR isoforms was increased following infection with MHV68, expression of LXR target genes was assessed next. mRNA levels were examined at 24 h postinfection, when peak mRNA levels of both LXR isoforms are observed (Fig. 1F) and just prior to the appearance of detectable LXR-dependent effects on viral DNA synthesis (Fig. 1C and D). Surprisingly, expression of LXR target genes was increased modestly, if at all, following infection. MHV68 infection induced a 1.8-fold increase in mRNA levels of ABCA1, a direct LXR target gene (Fig. 3A). Similarly, we observed a small (1.4-fold) increase in the mRNA levels of the acetyl-coenzyme A carboxylase (ACC) gene, another LXR target gene (Fig. 3B). When additional LXR target genes were examined, FADS2 mRNA levels were not changed by MHV68 infection (Fig. 3C), and mRNA levels of SCD2 were decreased in infected macrophages (Fig. 3D). Thus, increased expression of both LXR isoforms in MHV68-infected macrophages did not stimulate a corresponding increase in the expression of LXR target genes.

### LXRs suppress expression of target genes in primary macrophages.

Having observed an antiviral effect of LXRs in MHV68-infected macrophages alongside a modest (or nonexistent) increase in LXR target gene expression, we sought an unbiased approach to define the antiviral mechanism of LXRs. Affymetrix microarrays were used to analyze the effect of LXRs on global transcription after MVH68 infection.

**FIG 4:**
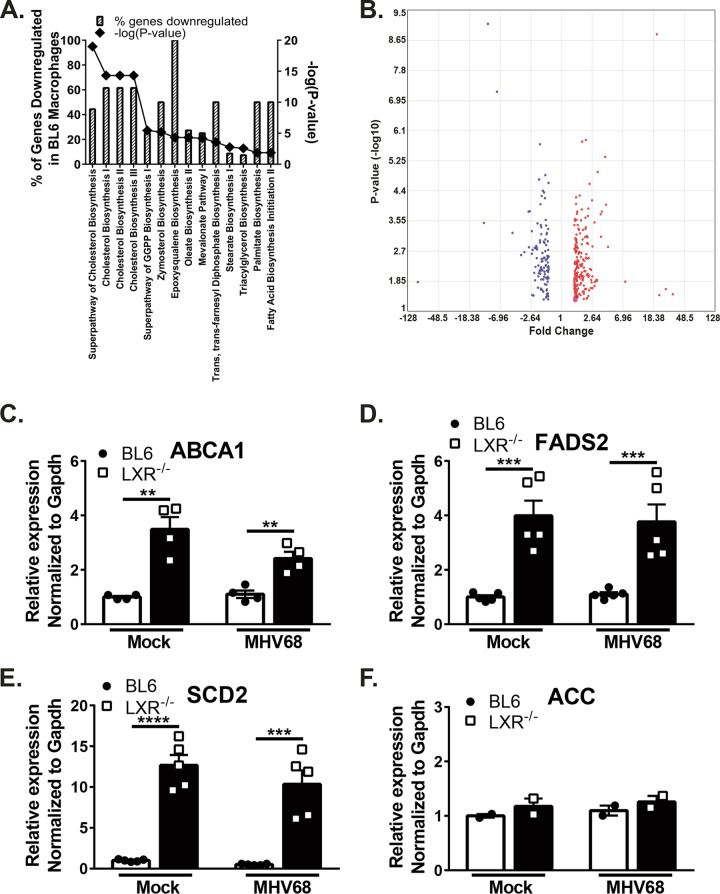
LXR deficiency results in increased expression of classical LXR target genes in primary macrophages. (A) Three independent batches of BL6 and LXR^−/−^ BMDM were infected at an MOI of 10 for 24 h. Total RNA was isolated, and relative gene expression levels were compared via Affymetrix microarray. Differentially expressed genes were analyzed by the use of Ingenuity Pathway Analysis software. Gray bars represent the percentages of genes in each statistically significant category for which the expression was lower in BL6 macrophages than in LXR^−/−^ macrophages. Diamonds indicate corresponding –Log data (*P* value) for each category. (B) Volcano plot representation of differentially expressed genes. (C to F) BMDM from BL6 and LXR^−/−^ mice were infected at an MOI of 5 for 24 h or were mock infected, with subsequent measurement of relative gene expression levels by qRT-PCR. Data are representative of results from two or more experiments.

Genes differentially expressed in LXR^−/−^ MHV68-infected macrophages compared to BL6 infected controls were analyzed to define pathways that are regulated by LXRs. Not surprisingly, most of the differentially expressed genes fell into the categories traditionally associated with LXR targets, such as cholesterol and fatty acid metabolism (Fig. 4A and B). However, in contrast to the classical role of LXRs in activating such genes, these LXR target genes were expressed to a greater level in the absence of LXRs.

To confirm and extend the conclusions of the microarray analyses, differential expression of LXR target genes was examined by quantitative reverse transcription-PCR (qRT-PCR) (see Table 2 in Text S1 in the supplemental material for side-by-side comparisons of microarray and qRT-PCR data). Consistent with the microarray results, ABCA1 mRNA levels were significantly elevated in MHV68-infected LXR^−/−^ macrophages compared to BL6 controls (Fig. 4C). A similar increase in ABCA1 mRNA levels was also observed in uninfected macrophages, suggesting that LXR-dependent suppression of target genes is an intrinsic metabolic mechanism that supports the profound antiviral nature of this innate immune cell type. Similarly, increased expression of FADS2 and SCD2 LXR target genes was found both at baseline and following infection of LXR^−/−^ macrophages (Fig. 4D and E). Interestingly, not every classical LXR target gene was expressed to a greater level in LXR^−/−^ macrophages. Specifically, expression of the ACC gene was not affected by LXR deficiency in primary macrophages (Fig. 4F). Thus, LXRs suppressed expression of select classical target genes in macrophages prior to and during MHV68 infection.

10.1128/mBio.01115-18.1 TEXT S1  Sequences of primers used to measure mRNA levels of indicated genes by qRT-PCR (Table 1) and fold changes in expression of indicated genes between infected BL6 and LXR^−/−^ macrophages as determined by microarray and qRT-PCR approaches (Table 2). Download TEXT S1, DOCX file, 0.01 MB. Copyright © 2018 Lange et al. 2018 Lange et al. This content is distributed under the terms of the Creative Commons Attribution 4.0 International license.

### LXRs do not regulate MHV68 replication in mouse embryonic fibroblasts (MEFs).

**FIG 5:**
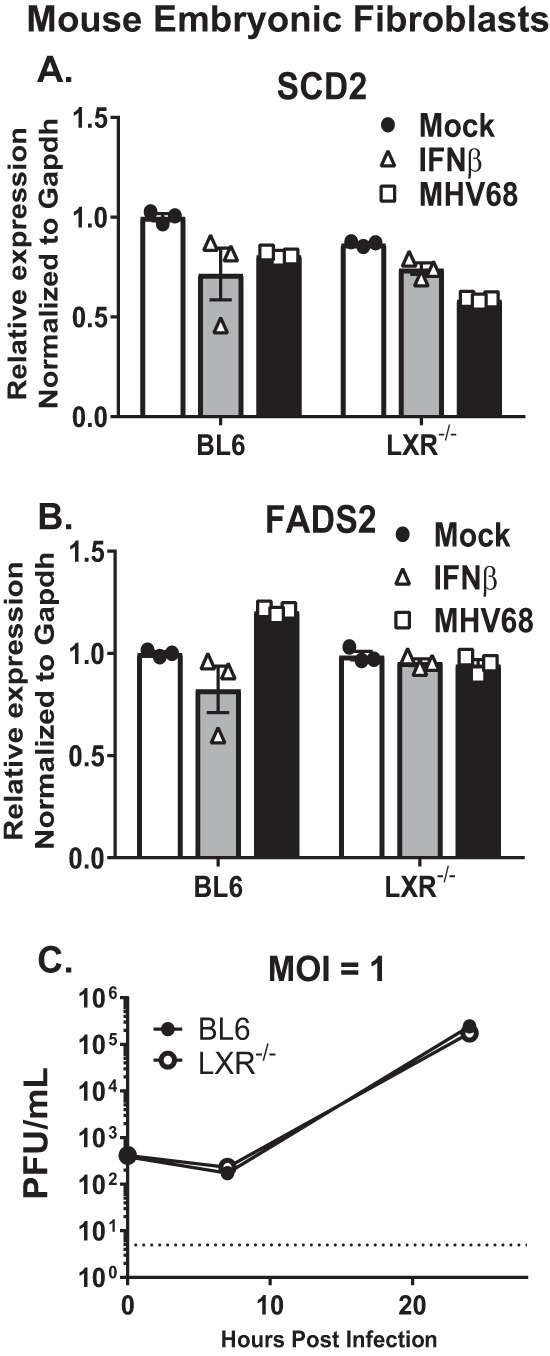
LXRs do not regulate MHV68 replication in mouse embryonic fibroblasts (MEFs). MEFs were prepared from BL6 embryos or embryos genetically deficient in both LXR isoforms (LXR^−/−^) and were mock treated, infected with MHV68 (MOI = 1), or treated with exogenous IFN-β. (A and B) MRNA levels of indicated genes were measured at 24 h postinfection/treatment by qRT-PCR for SCD2 (A) or FADS2 (B). (C) Viral titers were determined at the indicated times postinfection. Data are representative of results from two or more experiments.

Macrophages are critical players of the innate immune response that encounter viral pathogens at the onset of infection. It is not surprising that these immune cells may have evolved a mechanism to constitutively attenuate activity of metabolic pathways that benefit invading pathogens. To determine the extent to which LXR-mediated suppression of lipid synthesis genes and viral replication also operates in nonimmune cells, gene expression and MHV68 infection was examined in MEFs derived from BL6 or LXR^−/−^ mice. In contrast to that observed in primary macrophages, genetic deficiency of both LXR isoforms did not result in increased transcription of LXR target genes in MEFs (Fig. 5A and B). Further, the levels of MHV68 replication were similar in BL6 and LXR^−/−^ MEFs (Fig. 5C). Thus, antiviral effects of LXRs were selective for innate immune cells.

### Treatment with artificial LXR agonists reactivates expression of LXR target genes in MHV68-infected macrophages but does not increase MHV68 replication.

**FIG 6:**
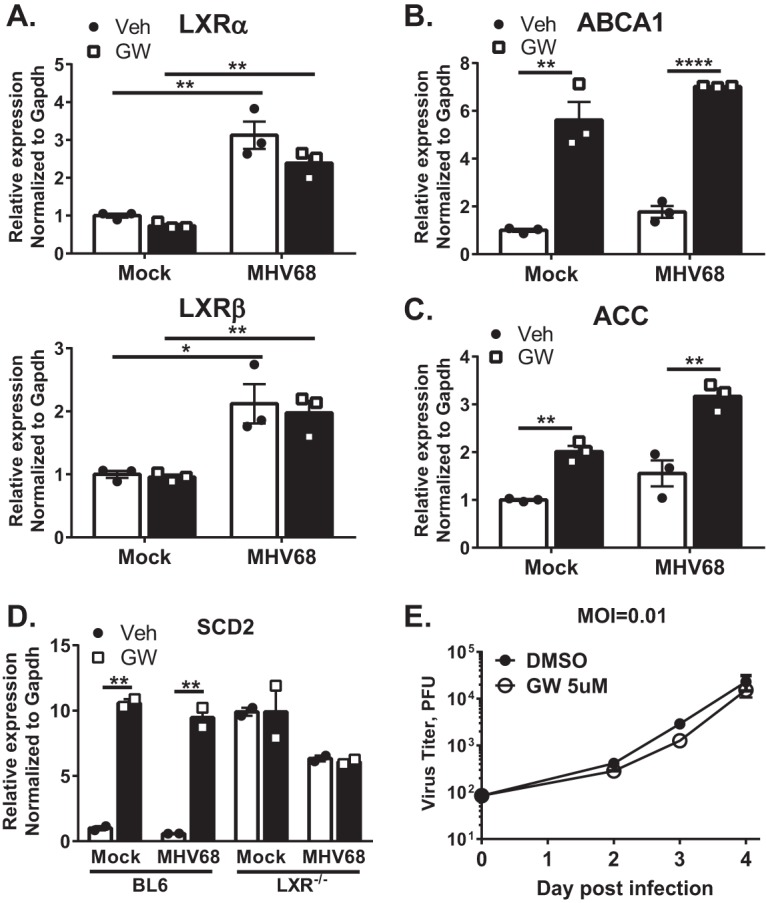
Treatment with artificial LXR agonists promotes expression of LXR target genes but does not increase MHV68 replication. (A to C) BL6 BMDM were infected at an MOI of 5 or mock infected. At 20 h postinfection, macrophages were treated with 5 µM GW3965 (GW) or vehicle control (Veh) for 4 h, followed by isolation of total RNA. qRT-PCR was performed to determine the relative expression levels of the indicated genes. (D) Experimental parameters were identical to those described for panels A to C with both BL6 and LXR^−/−^ macrophages used. (E) BL6 BMDM were infected at an MOI of 0.01 and treated with 5 µM GW3965 or vehicle control. Viral titers were determined at the indicated times. Data shown are representative results from at least two independent experiments.

Having observed LXR-mediated repression of target genes in macrophages, we next wanted to determine the extent to which this repression is reversible. Mock- and MHV68-infected BL6 macrophages were treated with GW3965 (GW), a potent synthetic agonist of both LXR isoforms (21). GW treatment did not alter mRNA levels of either LXR isoform (Fig. 6A), indicating that LXRs may not increase their own expression in mouse macrophages. As expected, treatment of mock-infected macrophages with GW increased the mRNA levels of the ABCA1, ACC, and SCD2 LXR target genes (Fig. 6B to D). The GW-dependent increase in SCD2 gene expression did not occur in LXR^−/−^ macrophages (Fig. 6D), indicating the specificity of the GW compound in the regulation of this particular LXR target gene. Importantly, GW treatment also increased expression of LXR target genes in MHV68-infected macrophages (Fig. 6B to D), indicating that LXR-mediated repression of target genes can be reversed by a potent synthetic agonist.

Having observed increased expression of LXR target genes in macrophages treated with the synthetic agonist, we wanted to test the extent to which such treatment alters MHV68 replication. Interestingly, GW treatment of infected macrophages had no effect on MHV68 replication, in contrast to increased viral replication in LXR^−/−^ macrophages (compare Fig. 6E and 1A). Thus, pan-activation of LXRs with the synthetic agonists, while increasing expression of LXR-dependent genes, did not promote MHV68 replication.

### The activity of fatty acid and cholesterol synthesis pathways is increased in LXR-deficient macrophages.

**FIG 7:**
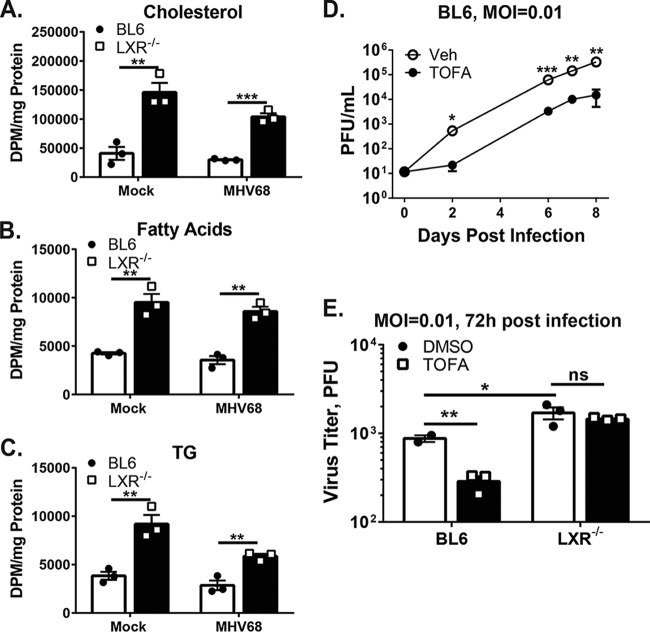
Activity of fatty acid and cholesterol synthesis pathways is increased and antiviral effects of fatty acid synthesis inhibition are attenuated in LXR^−/−^ macrophages. (A to C) BMDM of the indicated genotypes were infected at an MOI of 5 or mock infected. Macrophages were exposed to 5 µCi [^3^H]acetic acid for 8 h (16 to 24 h postinfection). At 24 h postinfection, macrophages were harvested; cholesterol (A), fatty acids (B), and triglycerides (C) data were resolved by TLC; and incorporation of ^3^H label was quantified by scintillation counting. DPM, disintegration per minute. (D and E) BMDM of indicated genotypes were infected at an MOI of 0.01 and treated with 10 µg/ml TOFA or vehicle control. Viral titers were measured at the indicated times postinfection. Data are representative of results from at least two independent experiments.

Having observed elevated gene expression of cholesterol and fatty acid synthesis enzymes in the absence of LXRs, we wanted to assess the extent to which this increased gene expression altered the metabolic activity of fatty acid and cholesterol synthesis pathways. The activity of these pathways was assessed by measuring the incorporation of [^3^H]-acetic acid into products of the two synthesis pathways. Infected LXR^−/−^ macrophages exhibited increased incorporation of the radioactive acetic acid into cholesterol (3.5-fold), fatty acids (2.4-fold), and triglycerides (2.1-fold) compared to infected BL6 macrophages (Fig. 7A to C). These elevated levels of synthesis were also present in mock-infected LXR^−/−^ macrophages (cholesterol, 3.6-fold; fatty acids, 2.2-fold; triglycerides, 2.4-fold), consistent with the idea that LXR-dependent suppression of lipid synthesis pathways contributes to an intrinsic antiviral state of macrophages.

### Antiviral effects of fatty acid synthesis inhibition are attenuated in LXR^−/−^ macrophages.

The fatty acid synthesis pathway supports latent and lytic KSHV infection (11, 12). Treatment of primary macrophages with the inhibitor of the fatty acid synthesis pathway (TOFA [tetradecyloxy-2-furoic acid]) also attenuated MHV68 replication (Fig. 7D), indicating that the reliance on fatty acid synthesis is a common feature of gammaherpesvirus infection.

Having observed higher activity of fatty acid synthesis in LXR^−/−^ macrophages (Fig. 7B and C), we wanted to test the hypothesis that these macrophages are less susceptible to antiviral effects of fatty acid synthesis inhibitors. Treatment of BL6 macrophages with TOFA attenuated MHV68 replication compared to the results seen with a vehicle-treated control (Fig. 7E). In contrast, the levels of MHV68 replication were similar in LXR^−/−^ macrophages treated with vehicle or TOFA. Thus, higher activity of fatty acid synthesis pathway in the absence of LXRs rendered MHV68 replication less susceptible to synthetic inhibitors of this pathway.

## DISCUSSION

**FIG 8:**
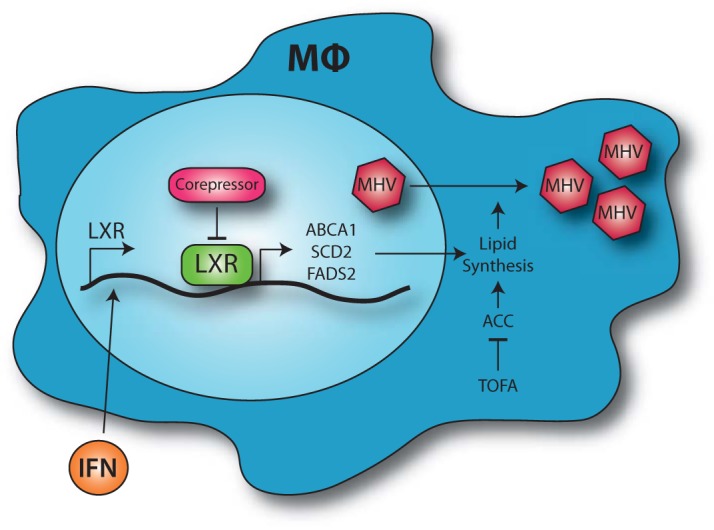
Working model. Macrophages respond to type I IFN produced during viral infection by increasing expression of LXRs. The LXRs interact with nuclear corepressors to limit the expression of select LXR target genes. This repression of gene expression limits the activity of the proviral cholesterol and fatty acid synthesis pathways and restricts MHV68 replication.

We show that LXR-mediated suppression of lipid synthesis pathways imposes an intrinsic immune state that restricts gammaherpesvirus replication in primary macrophages, representing a critical cell type of the innate immune system. Our results support the following working model (Fig. 8). Consistent with the antiviral nature of LXRs in macrophages, type I IFN produced by these cells following viral infection further increases baseline expression of both LXR isoforms. However, in contrast to their classical role as transcriptional activators, LXRs repress select target genes, decreasing expression of enzymes mediating fatty acid synthesis and cholesterol efflux proteins. As a result of this transcriptional repression of LXR target genes, there is attenuated activity of both the fatty acid and cholesterol synthesis pathways, two biosynthetic pathways that support MHV68 replication.

### Regulation of LXR target genes in infected cells.

This study demonstrated that gene repression, rather than activation, may be the biologically relevant antiviral role of LXRs in primary innate immune cells. This is consistent with a previous report by Wagner et al. (22), which additionally emphasized the cell type specificity of this phenomenon, as LXR-mediated gene repression was not detected in liver, muscle, or fibroblasts. We have found that LXRs did not mediate antiviral effects in MEFs (Fig. 5). In contrast, our observation that LXR-mediated gene repression is antiviral in primary macrophages highlights a previously unappreciated mechanism by which innate immune cells maintain a primed, intrinsic antiviral state even prior to the encounter with the pathogen.

In their unliganded state, nuclear receptors, including LXRs, recruit nuclear corepressors (NCoRs), primarily NCoR1 and NCoR2 (22, 23). The NCoRs then facilitate transcriptional repression through the recruitment of histone deacetylases to the target gene promoters. It is possible that the LXR complex delivers the nuclear corepressors to the corresponding promoters, as expression of LXR-dependent genes increases in macrophages genetically deficient in both isoforms (Fig. 4). It is not clear what drives expression of these genes in the absence of LXRs, including during infection, when a viral factor may enact a "tug of war" with LXRs to increase expression of target genes. The ABCA1 promoter contains functional binding sites for upstream stimulatory factor 1 (USF1) and USF2 (22), with the possibility that USF1/USF2 may contribute to ABCA1 expression in LXR^−/−^ macrophages. Interestingly, varicella-zoster virus and Epstein-Barr virus (EBV), which are alpha- and gammaherpesviruses, respectively, usurp USF1 and USF2 to facilitate expression of viral lytic transactivators (24–26). Furthermore, USF1 binds to the latency-associated transcript promoter of herpes simplex virus (27) and activates the EBV DNA polymerase gene promoter (28). Finally, the E6 protein of human papillomavirus activates telomerase expression by inhibiting the USF-mediated repression of tert (29). Future studies will determine the relative contributions of USF and other host and viral transcription factors to the expression of LXR target genes during gammaherpesvirus infection.

Additional host factors may enforce repression of LXR target genes. IRF-3 was shown to attenuate LXR activity, as demonstrated by an IRF-3-dependent reduction in LXR target gene expression following treatment with Toll-like receptor 3 (TLR3) and TLR4 ligands (30, 31). MHV68 infection activates IRF-3, which supports type I IFN expression by infected macrophages (17). However, we observed similar levels of expression of LXR target genes in BL6 and IRF-3^−/−^ macrophages (data not shown), suggesting that IRF-3 activation in the context of gammaherpesvirus infection does not further attenuate expression of LXR-driven genes.

### Regulation of virus replication by LXRs.

This report defines an underappreciated function of LXRs within primary immune cells that supports the intrinsic antiviral state of these cells. Specifically, LXRs limited the expression of genes that facilitate fatty acid and cholesterol synthesis, pathways which promote replication of diverse viruses. While the mechanism by which inhibition of fatty acid synthesis restricts gammaherpesvirus replication is unknown, the Lagunoff group demonstrated that KSHV requires this pathway very late in its replication cycle, likely to facilitate viral egress (12). Attenuation of cholesterol biosynthesis restricts MHV68 replication; however, this restriction is not due to suboptimal cholesterol availability. Instead, MHV68 replication is supported by isoprenoids, intermediates of the cholesterol synthesis pathway that are required for protein prenylation (13). Those previous reports, as well as data presented in this study, support the hypothesis that the LXR-mediated repression of cholesterol and fatty acid synthesis in primary macrophages is responsible for the antiviral activity of LXRs during MHV68 infection.

While this antiviral mechanism likely applies to other gammaherpesviruses, and possibly to other DNA viruses, there remain many questions regarding the role of LXR in RNA virus replication. Until this report, most of the studies examining the role of LXRs during viral infection were performed in the context of LXR stimulation performed with artificial LXR agonists. In the context of HCV, HIV, and NDV infections, LXR activation attenuated viral replication in an ABCA1-dependent manner, suggesting that these viruses rely on cellular cholesterol for optimal replication (7, 9, 32, 33). Another report suggested that the LXR-mediated attenuation of HCV replication is due to the increase in LXR-dependent expression of the inducible degrader of the LDL receptor, thus limiting uptake of extracellular cholesterol (8).

Interestingly, treatment of MHV68-infected macrophages with a synthetic LXR agonist did not confer the expected proviral effect, in spite of induction of fatty acid and cholesterol synthesis genes (Fig. 5). It is possible that the off-target effects of this synthetic LXR agonist counteracted the proviral effects on fatty acid and cholesterol synthesis. Such off-target effects could have contributed to altered replication of RNA viruses in synthetic agonist-treated cells. Alternatively, synthetic agonists may induce LXR activation that is not entirely physiological. We have observed that some genes, such as the ACC gene, that were expressed at similar levels in LXR-sufficient and LXR-deficient macrophages (Fig. 4F) were robustly induced by the synthetic agonist (Fig. 6C). This suggests that activation of LXR with synthetic agonists may not faithfully represent physiological regulation of LXR-dependent genes.

In this study, we also tested the contribution of CH25H expression to the antiviral activity of LXRs. 25HC, a product of CH25H, exerts broad antiviral functions (19) and is an agonist of LXRs (14). We have shown that CH25H expression specifically represses late stages of MHV68 DNA synthesis without affecting expression of type I IFN and interferon-stimulated genes in infected primary macrophages (15). Interestingly, similarly to what was previously observed for mouse cytomegalovirus, the antiviral activity of 25HC was independent of LXR expression in MHV68-infected macrophages (Fig. 2). Thus, the fascinating mechanisms underlying antiviral activity of 25HC are clearly LXR independent.

In summary, there is a growing appreciation of the interplay between viral infection and host cell metabolism, including fatty acid and cholesterol synthesis pathways (11–13, 19, 34–39). This interplay is likely to be modified by specific viruses and the cell types targeted for infection. An important issue to be resolved is that of elucidating the exact molecular mechanism by which lipogenic pathways facilitate viral replication. While synthesis of lipids and cholesterol may facilitate structural features of virions and support the cellular membrane remodeling observed in some infections, it is likely that there are several metabolites of lipogenic pathways that alter the signaling milieu of the infected cell to facilitate viral replication. We found that the isoprenoid intermediates produced by the cholesterol synthesis pathway, and not cholesterol *per se*, are what support gammaherpesvirus replication in macrophages (13). Thus, prenylation of cellular and/or viral proteins using isoprenoid intermediates is likely at the heart of the proviral activity of cholesterol synthesis pathway during gammaherpesvirus replication. Finally, because novel LXR agonists are being developed to treat cardiovascular diseases, it is important to consider potential effects of such future therapies on the ubiquitous herpesvirus infections in humans.

## MATERIALS AND METHODS

Additional details are available in Table S1 in the supplemental material.

10.1128/mBio.01115-18.2 TABLE S1  Additional details of the genes discussed. Download TABLE S1, XLSX file, 0.03 MB. Copyright © 2018 Lange et al. 2018 Lange et al. This content is distributed under the terms of the Creative Commons Attribution 4.0 International license.

### Ethics statement.

All experimental manipulations of mice were approved by the Institutional Animal Care and Use Committee of the Medical College of Wisconsin (AUA971) and adhered to the recommendations of the Guide for the Care and Use of Laboratory Animals of the National Institutes of Health and the American Veterinary Medical Association Guidelines on Euthanasia.

### Animals and primary cell cultures.

All mouse strains used in this study were maintained at the Medical College of Wisconsin (MCW) animal facility. C57BL/6J mice (referred to as BL6 mice) and CH25H^−/−^ mice (40) were originally purchased from Jackson Laboratories (Bar Harbor, ME). LXRα^−/−^ mice and LXRβ^−/−^ mice (41) (both C57BL/6 genetic background) were kind gifts of Qing Miao (MCW) and Peter Tontonoz (University of California, Los Angeles [UCLA]), respectively. Mice lacking individual LXR isoforms were crossed and doubly deficient mice produced via breeding LXRβ^+/−^ LXRα^−/−^ parents. IFNAR1^−/−^ mice on the C57BL/6 genetic background were a gift from Mitchell Grayson (42). Bone marrow was harvested from male and female mice that were between 3 and 10 weeks of age. Primary bone marrow-derived macrophages were generated as previously described (43). At least two independently derived batches of macrophages were used in each experiment. MEFs were generated from embryonic day 12 (E12) to E14 embryos. For LXR^−/−^ MEFs, each embryo was individually homogenized after dissection; passage 1 MEFs derived from individual embryos were genotyped to identify fibroblasts lacking both LXR isoforms. BL6 MEFs were pooled at early passages.

### Virus infection and cell treatment.

Wild-type MHV68 viral stocks were prepared and titers determined on NIH 3T12 cells. Bone marrow-derived macrophages or MEFs were infected with live or UV-inactivated MHV68 at the indicated multiplicity of infection (MOI) for 1 h to allow adsorption and were washed 2 to 3 times with phosphate-buffered saline (PBS) prior to medium replenishment. UV inactivation of MHV68 was performed as previously described (17). Supplementation experiments were performed by adding the indicated compound(s) to the replenishment media following viral adsorption. GW3965 and TOFA [tetradecyloxy-2-furoic acid] were purchased from Cayman Chemical (Ann Arbor, MI). GW3965 was dissolved in dimethyl sulfoxide (DMSO) and then diluted in cell culture media to a concentration of 5 µM. TOFA was dissolved in DMSO and then diluted in cell culture media to a concentration of 10 µg/ml. Control cell cultures were treated with equivalent amounts of DMSO.

### qRT-PCR and viral DNA synthesis.

To quantify the relative abundances of mRNA transcripts, total RNA was harvested from infected cells using Trizol according to the instructions of the manufacturer (Invitrogen, Carlsbad, CA). One microgram of RNA was treated with RNase-free DNase (Ambion, Austin, TX) and split into two halves. The first half was subjected to reverse transcription using Moloney murine leukemia virus (M-MLV) reverse transcriptase (Invitrogen, Carlsbad, CA) and oligo(dT) primers. The second DNase-treated RNA half was subjected to mock reverse transcription in the absence of the enzyme (−RT control). cDNA, including the –RT controls, was diluted 3-fold and measured, in triplicate for each dilution, by real-time PCR using an iQ5 multicolor real-time PCR detection system (Bio-Rad, Hercules, CA). Gene-specific cDNA was amplified using the probes listed in Table 1 in Text S1 in the supplemental material. All primers were evaluated for specificity and used within the corresponding linear range for each primer pair. The delta-delta threshold cycle (*C*_*T*_) method was used to quantify the relative abundance of each cDNA.

Measurement of viral DNA synthesis was performed as follows. Infected cells were washed with PBS and lysed in a buffer containing 10 mM Tris-HCl, 1 mM EDTA, 0.8% SDS, and 20 µg/ml of proteinase K (Sigma-Aldrich, St. Louis, MO). Following overnight protein digestion at 56°C, DNA was extracted with phenol-chloroform and precipitated using a standard sodium acetate-ethanol treatment. The DNA pellet was resuspended in TE buffer (10 mM Tris-Cl [pH 8], 1 mM EDTA), and viral DNA was measured by real-time PCR using core gene 50 promoter primers (44) and normalized to corresponding levels of GAPDH (glyceraldehyde-3-phosphate dehydrogenase).

### Lipid synthesis.

Macrophages infected for 24 h, or mock infected, were labeled with 5 µCi ^3^H acetic acid (PerkinElmer, Waltham, MA) for 8 h and then washed twice with cold PBS. Lipids were extracted by adding 1.5 ml of isopropanol and incubating the cell culture dishes overnight at −20°C. The isopropanol was then collected into glass screw-cap tubes. An additional 1.5 ml of isopropanol was added to the cell culture dishes, and the reaction mixture was again incubated overnight at −20°C. The isopropanol was collected and pooled with the initial 1.5 ml, for a total of 3 ml. The cellular proteins were solubilized in 500 µl of 0.1 N NaOH on a plate rocker for 30 min at room temperature. The protein concentrations were quantified using the Lowry method. The extracted lipids were dried under a gentle flow of N_2_ at 50°C and then dissolved in dichloromethane containing a lipid mixture of known standards to aid in visualization of lipid bands. The mixture consisted of cholesterol (30 µl, 1 mg/ml), palmitate (20 µl, 10 mg/ml), triglyceride (15 µl, 10 mg/ml), and cholesteryl ester (5 µl, 10 mg/ml) standards. The entire volume of the lipid solution was spotted onto high-performance thin-layer chromatography (HP-TLC) silica gel plates (Millipore, Billerica, MA) and allowed to dry. Lipids were separated by a solvent system containing hexane-isopropyl ether-acetic acid (65:35:2 [vol/vol/vol]). Lipids were then visualized by exposure to iodine, the desired bands were scraped from the plate, and ^3^H incorporation into each lipid band was quantified by liquid scintillation counting (Packard Tri-Carb 2100 TR liquid scintillation counter; PerkinElmer, Waltham, MA). The incorporation of the radiolabel was then normalized to the cellular protein concentration for each sample.

### Western blot analysis.

The antibodies used were anti-β-actin (Novus Biological, Littleton, CO) (1:20,000), anti-LXRα (Abcam, Inc., Cambridge, MA) (1:1,000), and a secondary goat anti-mouse or anti-rabbit horseradish peroxidase (HRP)-conjugated secondary antibody (Jackson ImmunoResearch, Inc., West Grove, PA) (1:25,000).

### Microarray and analyses.

Three independent batches of BL6 (wild-type) and LXR^−/−^ bone marrow-derived macrophages were infected in triplicate at an MOI of 10 for 24 h. Total RNA was isolated as described above and submitted to the Genomics Core Facility at Brown University for microarray analysis. Total RNA (100 ng) was analyzed for its integrity using an Agilent BioAnalyzer RNA6000 Nano chip. The purity of the samples was assessed using an ND100 NanoDrop spectrophotometer. All samples had RNA integrity numbers (RINs) of >8.8 and 260/280 ratios of >1.8. Total RNA (100 ng per sample) was used in the initial reverse transcription reaction and subsequent *in vitro* transcription (IVT) reaction followed by amplification overnight. IVT bead-purified cRNA (15 µg) was reverse transcribed and dUTP labeled, and the resulting cDNA was bead purified. cDNA (5.5 µg) was fragmented and end labeled. Approximately 2.4 µg hybridized to Affymetrix Mouse Gene St 1.0 arrays overnight. Hybridized arrays were washed and stained using an Affymetrix FS 450 Fluidics Station with Fluidics Script FS450-0007 before being visualized using an Affymetrix 3000 7G scanner. All steps of the amplification protocol and thermocycler settings were carried out according to the manufacturer’s instructions, which can be found in the WT Plus Reagent kit (Manual GeneChip Whole Transcript). The resulting .cel files were uploaded to Partek Genomics Suite version 6.6. An up- or downregulation of at least 1.5-fold with a false-discovery-rate (FDR)-corrected *P* value of 0.05 was set as the threshold in the analyses for differentially regulated transcripts.

### Statistical analyses.

Statistical analyses were performed using Student’s *t* test (Prism, GraphPad Software, Inc.).
