# Supplementary material for: Liver X Receptors Suppress Activity of Cholesterol and Fatty Acid Synthesis Pathways To Oppose Gammaherpesvirus Replication
Source: mBio. 2018 Jul 17;9(4):e01115-18. doi: 10.1128/mBio.01115-18 (PMC6050960; doi:10.1128/mBio.01115-18)
Supplement: TEXT S1 [file mbo004183983s2.docx]

**Supplemental Tables**.

**Table 1. Primers used for qRT-PCR analyses of gene expression**

| **Gene** | **Forward (5'-3')** | **Reverse (5'-3')** |
| --- | --- | --- |
| LXRα | TAAGGGAGAGTCAACAGG | GGTCAACAAGGTCTTCAG |
| LXRβ | GCAGTTGGCACTAGAAG | GGTAGGCTGAGGTGTAA |
| ABCA1 | GTTTCAGAGCAGATTTCTAAG | GGACTGTCACAGCTTTAT |
| ACC | GGAGCACCTCAAGCAGATATT | GGCTCTGACTTCTCCGTATTG |
| FADS2 | TCTCAGATCACCGAGGACTT | GGACAGGAGGAGAAAGAAGAAC |
| SCD2 | ACCTTCCTCACTCAGAGATACA | AAGCCTGGGAGGGATAAGA |
| ORF50 | AGAAACCCACAGCTCGCACTT | CAATATGCTGGACAGGCGTATC |

**Table 2. Comparison of gene expression changes analyzed with microarray vs. qRT-PCR approaches.**

| **Gene** | **Microarray Fold Change (Infected LXR^-/-^ v. Infected BL6)** | **qRT-PCR Fold Change (Infected LXR-/- v. Infected BL6)** |
| --- | --- | --- |
| **ABCA1** | 2.08 | 2.19 |
| **ACC** | 1.33 | 1.15 |
| **FADS2** | 3.8 | 3.41 |
| **SCD2** | 19.5 | 20.42 |
